# Idiosyncratic multisensory reweighting as the common cause for motion sickness susceptibility and adaptation to postural perturbation

**DOI:** 10.1371/journal.pone.0260863

**Published:** 2021-12-09

**Authors:** Merrick Dida, Corinne Cian, Pierre-Alain Barraud, Michel Guerraz, Rafael Laboissière

**Affiliations:** 1 Université Grenoble Alpes, Université Savoie Mont Blanc, CNRS LPNC UMR 5105, Grenoble, France; 2 Institut de Recherche Biomédicale des Armées, Brétigny sur Orge, France; 3 Université Grenoble Alpes, CNRS, CHU Grenoble-Alpes, Grenoble INP, TIMC-IMAG, Grenoble, France; University of Minnesota, UNITED STATES

## Abstract

Numerous empirical and modeling studies have been done to find a relationship between postural stability and the susceptibility to motion sickness (MS). However, while the demonstration of a causal relationship between postural stability and the susceptibility to MS is still lacking, recent studies suggest that motion sick individuals have genuine deficits in selecting and reweighting multimodal sensory information. Here we investigate how the adaptation to changing postural situations develops and how the dynamics in multisensory integration is modulated on an individual basis along with MS susceptibility. We used a postural task in which participants stood on a posturographic platform with either eyes open (EO) or eyes closed (EC) during three minutes. The platform was static during the first minute (baseline phase), oscillated harmonically during the second minute (perturbation phase) and returned to its steady state for the third minute (return phase). Principal component (PC) analysis was applied to the sequence of short-term power density spectra of the antero-posterior position of the center of pressure. Results showed that the less motion-sick a participant is, the more similar is his balance between high and low frequencies for EO and EC conditions (as calculated from the eigenvector of the first PC). By fitting exponential decay models to the first PC score in the return phase, we estimated, for each participant in each condition, the sluggishness to return to the baseline spectrum. We showed that the de-adaptation following platform oscillation depends on the susceptibility to MS. These results suggest that non motion-sick participants finely adjust their spectrum in the perturbation phase (i.e. reweighting) and therefore take longer to return to their initial postural control particularly with eyes closed. Thus, people have idiosyncratic ways of doing sensory reweighting for postural control, these processes being tied to MS susceptibility.

## Introduction

Motion sickness (MS) is a common disorder elicited by the abrupt body accelerations or repetitive movements (oscillations) that occur during passive transportation (as in cars, boats, trains and planes) [1, 2]. MS is usually triggered by a narrow band of body oscillation frequencies (from 0.08 to 0.4 Hz) [3]. This frequency band lies within the spectrum of spontaneous postural sway during natural stance and thus can cause postural instability through a type of wave interference [4]. Stoffregen and Riccio [5] suggested that this postural instability causes MS. According to an alternative “sensory conflict” or “neural mismatch” hypothesis, MS is caused by conflicts during the processing of multimodal sensory information about the individual’s motion relative to the environment [6–8]. A conflict occurs when the integrated sensory signals differ from previously recognized and stored motion paradigms. Vulnerability to MS has been linked to inability to adapt to the discrepancy between different sources of perceptual information [8]. In this second theoretical framework, postural instability is the consequence (and not the cause) of MS [9]. Even though these two theories are diametrically opposed, they both imply that postural dynamics reflect susceptibility to MS.

The results of several experimental studies support the hypothesis whereby the characteristics of postural control in the absence of motion are correlated with MS history for inertial motion. Differences in the sway path length have been observed when comparing individuals who suffered from MS with those who were never sick [10, 11]. The degree of MS susceptibility can also be predicted from the shape of the power spectral density (PSD) profile for spontaneous sway [10]. Laboissière et al. [10] found that the high frequency part of the sway signal was stronger in participants with a history of MS than in non-MS participants. These researchers suggested that the relative contributions of each sensory system involved in human stance (namely the visual, vestibular, and proprioceptive systems [12–14]), which are associated with different frequency bands of the sway signal) could explain individual differences in MS sensitivity. Stoffregen et al. [15] related the severity of seasickness to specific postural parameters measured before exposure to ship motion. These researchers performed a detrended fluctuation analysis of the temporal dynamics of body sway and showed that temporal self-similarity of the center of pressure (CoP) was higher in participants who experienced MS than in participants who did not experience MS. Varlet et al. [16] found that MS participants had a stronger coupling of their multi-axis postural oscillations with the complex oscillations induced by a ship at sea, relative to non-MS participants. These researchers suggested that low MS susceptibility was linked to the person’s ability to decouple the body sway from the ship oscillations and to use another sensory reference (i.e. the horizon, which is known to reduce the spatial magnitude of body sway at sea) [15, 17]. Furthermore, individuals with a high degree of habituation to seasickness show changes in their postural control strategy during exposure to motion [18]. This adaptive behavior might reflect the ability to select the appropriate sensory information for postural control [19]. This flexibility in postural adjustment might be: 1) linked to the “sensory reweighting” mechanism [20]; and 2) might be essential for reducing MS, regardless of the time interval between motion onset and MS onset.

According to the reweighting principle, the central nervous system prevents the loss of balance in situations of degraded sensory information by decreasing dependence on unreliable senses and giving more weight to reliable senses [21–29]. Sensory reweighting frameworks are commonly used to describe the dynamics of postural control. The single inverted pendulum model is a basic body model that involves sensory reweighting for postural control [28]. In this model, the human body (the inverted pendulum) has a single degree of freedom (the ankle angle) and a single motor output (muscle torque around the ankle). Information about body sway is conveyed to the central nervous system by the vestibular, visual and proprioceptive sensory systems. These three afferent estimations are weighted and then added together to yield a single estimation of the body sway, which is fed into a proportional integral derivative (PID) neural controller to produce the corrective ankle torque. The resulting spectrum of the body sway will therefore depend on the total feedback gain in the control loop, which is determined by the weights attributed to each sensory input and by the proportional gain of the PID controller. In undisturbed postural conditions, this model predicts that the body sway frequency spectrum will be essentially flat. However, when the feedback-loop gain is increased, a spectral peak at around 1 Hz appears, and the higher the gain, the higher the peak’s frequency. The gain increase in this model might be due to either the sum of all the sensory weights greater than one or to a gain increase in the proportional component of the PID controller. Hence, this model enables researchers to investigate sensory reweighting mechanisms.

Individuals may have idiosyncratic strategies for selecting and reweighting multimodal sensory information during postural control. Whenever appropriate postural adjustment to a new environmental situation is needed, failure to adapt (or the presence of a transient period with incorrect sensory weighting) can make the individual’s postural strategy ineffective. Thus, supposing a link between postural dynamics and MS, one can hypothesize than MS susceptibility is linked to an individual’s ability to reweight sensory cues for postural control in challenging situations. The present study addressed the relationship between susceptibility to MS and temporal aspects of postural regulation in general and transient periods of adaptation to new environmental conditions in particular. We tested postural adaptation using a postural task in which the participant stands on a platform with his/her eyes open (EO) or closed (EC) and had to counteract continuous oscillation applied to the platform. We determined how the spectral parameters of the body sway varied in response to changes in the participant’s visual and postural conditions and whether the time course of adaptation was correlated with MS susceptibility.

## Materials and methods

### Participants

27 women and 16 men participated in the experiment. Their mean age, height and weight were 23.2 years (SD = 4.05 years), 169 cm (SD = 8.81 cm), and 63.2 kg (SD = 11.7 kg), respectively. All of them had normal or corrected to normal vision and reported no history of neurological or musculoskeletal disorders that might affect their ability to maintain balance. All of the participants provided their written informed consent prior to initiation of the experiments. The study was performed in accordance with the ethical standards laid down in the 1964 Declaration of Helsinki and was approved by the local independent ethics committee of the University Grenoble-Alpes (IRB00010290-2017-07-04).

### Experimental setup

We assess our participants’ capability of dynamic sensory reweighting in a challenging postural control situation by making them stand on a custom-made force-measuring platform that detects the position of the center of pressure (CoP) on the horizontal plane. The experiment was performed with a custom-made device that provided servo-controlled motions of the platform (50 × 50 cm). The motor was controlled by a computer to generate anterior/posterior (AP) tilts of the support surface or to hold the device stationary in space. With regard to the position of the CoP, the mean precision for a 70 kg load applied on the center of the platform is inferior to 0.1 mm. Stimulus delivery and data collection were performed at a sampling frequency of 100 Hz. In order to prevent a fall in the case of imbalance, a frame, which did not touch the participant, surrounded the device.

### Behavioral experiments: Stimuli and procedure

**Fig 1:**
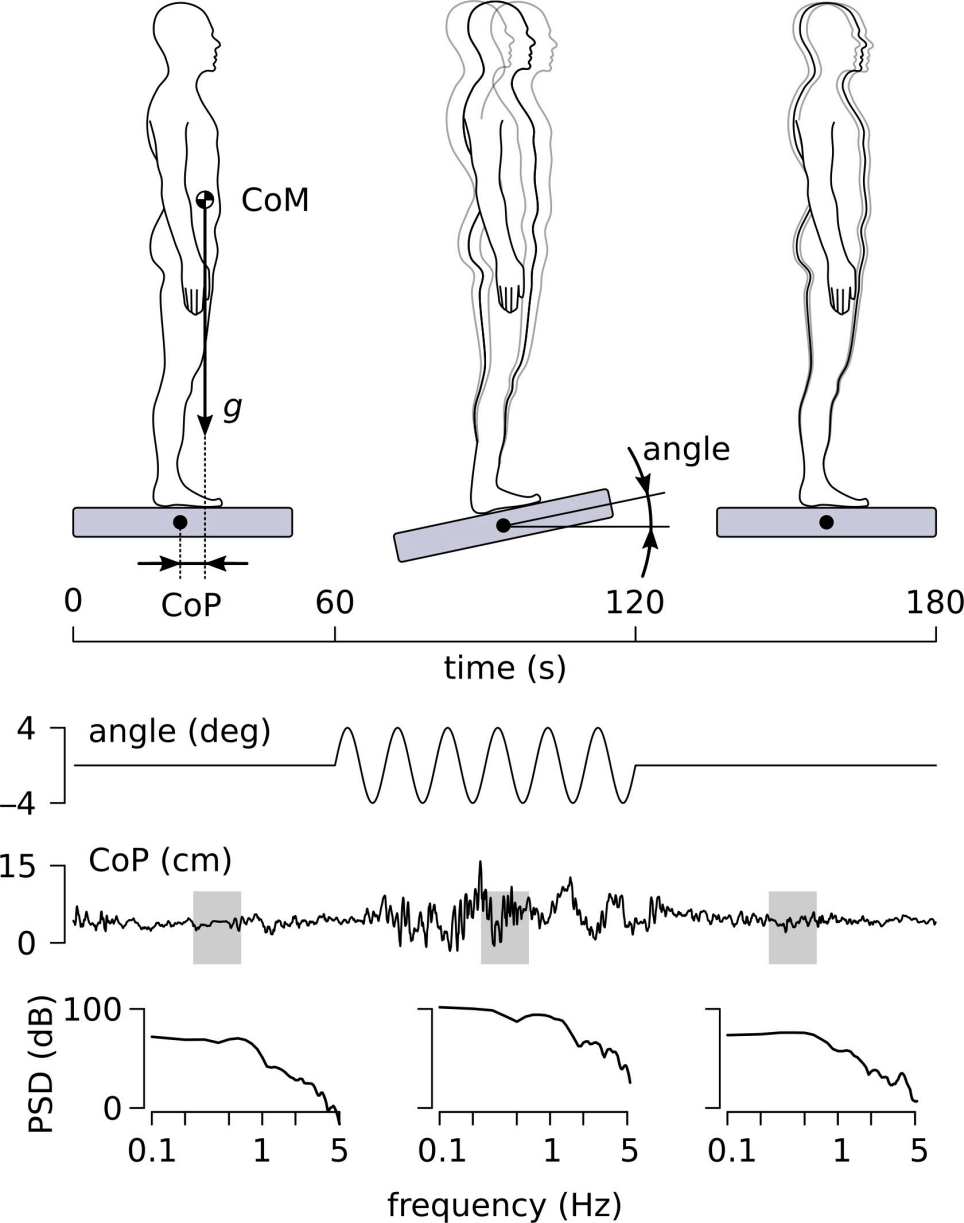
Experimental design and posturographic measurements. The three phases of the experiment (baseline, perturbation, and return) are shown in the upper panel. The time courses of the angle of the platform (for the specific case of 0.1 Hz) and of the anterior/posterior variation of the CoP are shown in the middle panel. Examples of the PSD for individual analysis windows in each phase, corresponding to the places indicated by gray rectangles, are shown in the bottom panel.

Prior to any data collection, the participants were informed about the general procedure and written informed consent was obtained. Thereafter, motion sickness susceptibilities were rated on the responses to a standardized questionnaire (Motion Sickness Susceptibility Questionnaire, MSSQ, [30]). The Motion Sickness Susceptibility score (MSS score) is defined as the sum of two subscores, the MSA (MS susceptibility before 12 years old) and the MSB (MS susceptibility during the last 10 years). For most subjects of our cohort, the periods corresponding to the MSA and the MSB scores overlapped. This could artificially increase the MSS score for those subjects [31]) and, therefore, only the MSB was used as MSS score. Then, the participant stood barefoot on the support surface, feet placed shoulder width apart, arms resting at their sides, with the rotation axis of the platform collinear with their ankle joints. Foot position was marked on the platform to ensure a consistent initial foot position within and across trials. They were asked to stand as still as possible, that is, avoiding to bend their knees or their hips or moving their arms and avoiding taking steps unless absolutely necessary. They wore headphones (Peltor optime III h540B with a 35 dB sound noise reduction) to limit auditory cues due to platform motion. The maximal sound intensity recorded at the level of the participant’s ears was 29 dB when the platform was static and 62 dB when the platform moved at 0.4Hz. The participants were presented with support surface tilts in the sagittal plane in two blocks corresponding to the two visual conditions, EC and EO. Each block consisted of three 180s trials corresponding to three oscillation frequencies of the supporting platform. During the measurement with EO, they were instructed to look straight ahead facing a poster representing horizontal and vertical crossing lines (distance from the eyes was 1.5m); “‘straight ahead” was defined as the visual field around eye level. With EC, they were asked to face forward as if they were looking straight ahead. The EC condition forces the participants to rely on vestibular and somatosensory information in order to overcome the perturbation and to keep their body in the vertical position. Each trial consisted of three phases depicted in Fig 1: 60 s with the platform still (baseline phase), 60 s with the platform oscillating sinusoidally about the medio-lateral axis (antero-posterior perturbation) with an amplitude of ±4 deg (perturbation phase), and 60 s with the platform still (return phase). During the perturbation phase, the platform oscillated sinusoidally at a constant frequency of 0.1, 0.2 or 0.4 Hz. All trials were followed by 1 minute of rest. There was also 5 minutes of rest between the different eye conditions. Half of the participants did the EO block first and the others started with EC. The order of the oscillation frequencies during each block was counterbalanced across participants.

After the experiment, the participants responded to the misery scale (MISC) questionnaire [32] Even though oscillations around 0.2 Hz are known to be particularly nauseogenic [33–36], none of our participants presented symptoms of MS during the experiment. The mean value of the MISC for all participants was 1.56 (in a scale from 0 to 10).

### Quantification and statistical analysis

#### Processing of postural data

**Fig 2:**
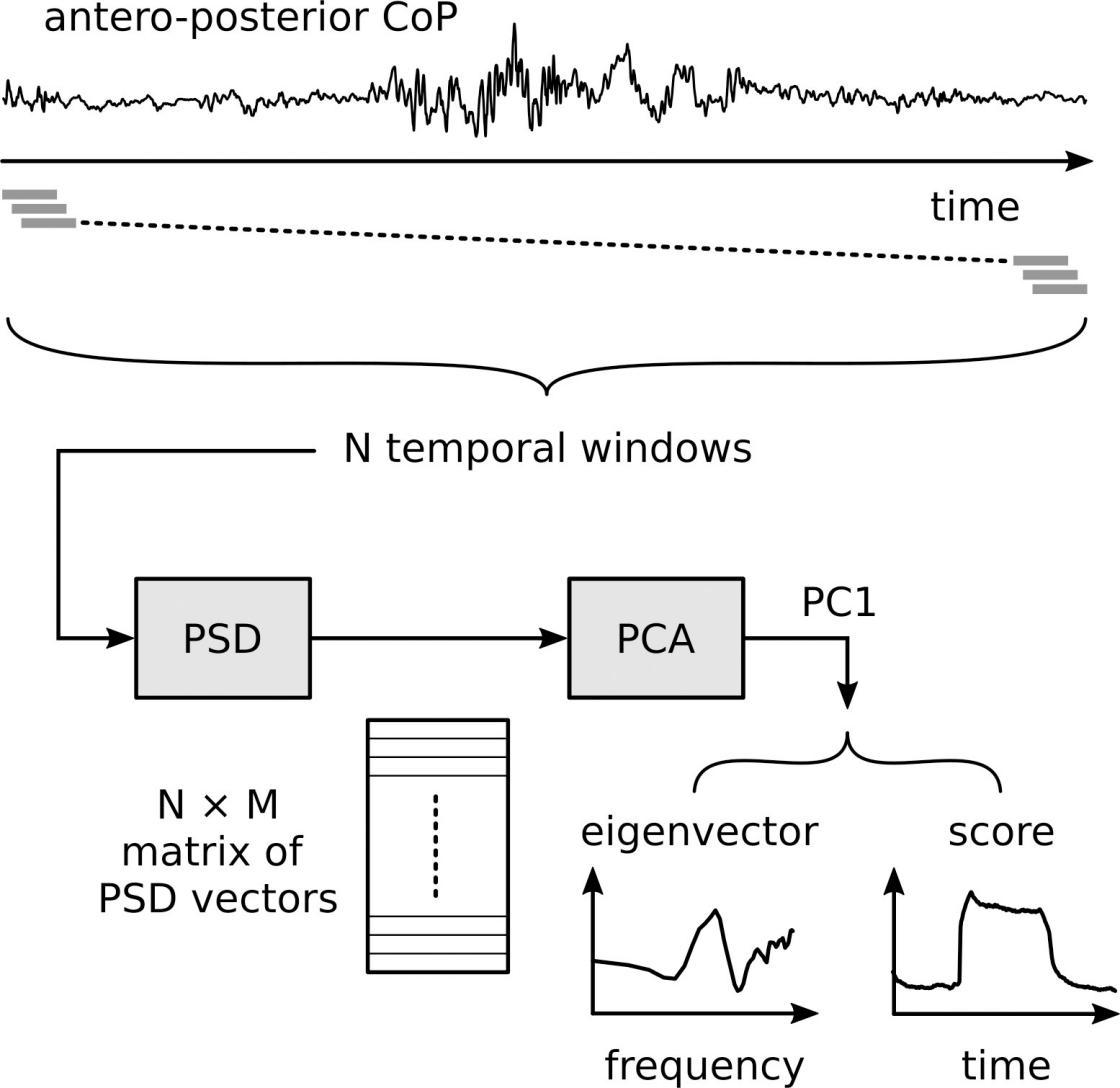
Depiction of the procedure for processing the posturographic signal. An example of the antero-posterior CoP signal covering 180 s is shown on the top. The sliding windows of 10 s are represented as gray rectangles just below the signals. The PSD analysis is done for each one of the *N* windows. The PSD vectors for the windows, which are vectors containing the *M* spectral components, are organized into an *N*×*M* matrix. This matrix is then fed to the PCA procedure. The first component PC1 is extracted and its eigenvector (spectral profile) and score (temporal profile) are used in the further analyses.

The whole procedure for extracting the dependent variables for the analyses from the posturographic signal is depicted in Fig 2 and is available from https://doi.org/10.17605/OSF.IO/BUK74. For each three-minute trial (258 trials, 43 participants × 2 visual conditions × 3 perturbation frequencies), we extracted the antero-posterior postural oscillations of the participant’s Centre of Pressure (CoP). In order to account for the spectro-temporal variations of the signal, we computed the power spectrum density (PSD) on a 10-seconds sliding window. The PSDs were computed for windows placed at every 10 ms, what corresponds to the sampling frequency of the posturographic signal. For further analysis, we limited the frequency range between 0 and 5 Hz. The choice of this maximum frequency was justified as we observed that the spectral amplitude dropped 60 dB between 0.01 Hz and 5 Hz, on average, across all conditions and participants (S1 Fig). Each PSD represents the spectrum at the time instant corresponding to the center of the window, such that, for instance, the window spanning from 0 to 10s yields the PSD for *t* = 5s. Each PSD is represented by a 50-dimensional vector, whose components are the amplitudes (in dB) of the PSD corresponding to the frequencies of the spectral analysis (from 0.1 to 5 Hz, in steps of 0.1 Hz). A principal component analysis (PCA) was then applied to this sequence of vectors for each trial. The first principal component (PC1), which is a temporal signal going from *t* = 5s to *t* = 165s, was used for further analysis. The PC1 explained 89% of the variance on average (minimum 71%, maximum 95%, see the distribution of this variance on the S2 Fig). For each trial, the eigenvector associated with the PC1 represents the main variation of the CoP spectrum during the three phases of the experiment. Neurophysiological models of postural control demonstrate the impact of the feedback control parameters on the CoP spectrum [7]. The PC1 score indicates how the CoP spectrum changes with time. In order to make results comparable across trials, the PC1 score was normalized, trial by trial, such that its mean value in the 30 to 55 s time interval was equal to 0 and its mean value in the time interval 90 to 115 s was equal to 1.

#### Spectral characteristics of the PC1

**Fig 3:**
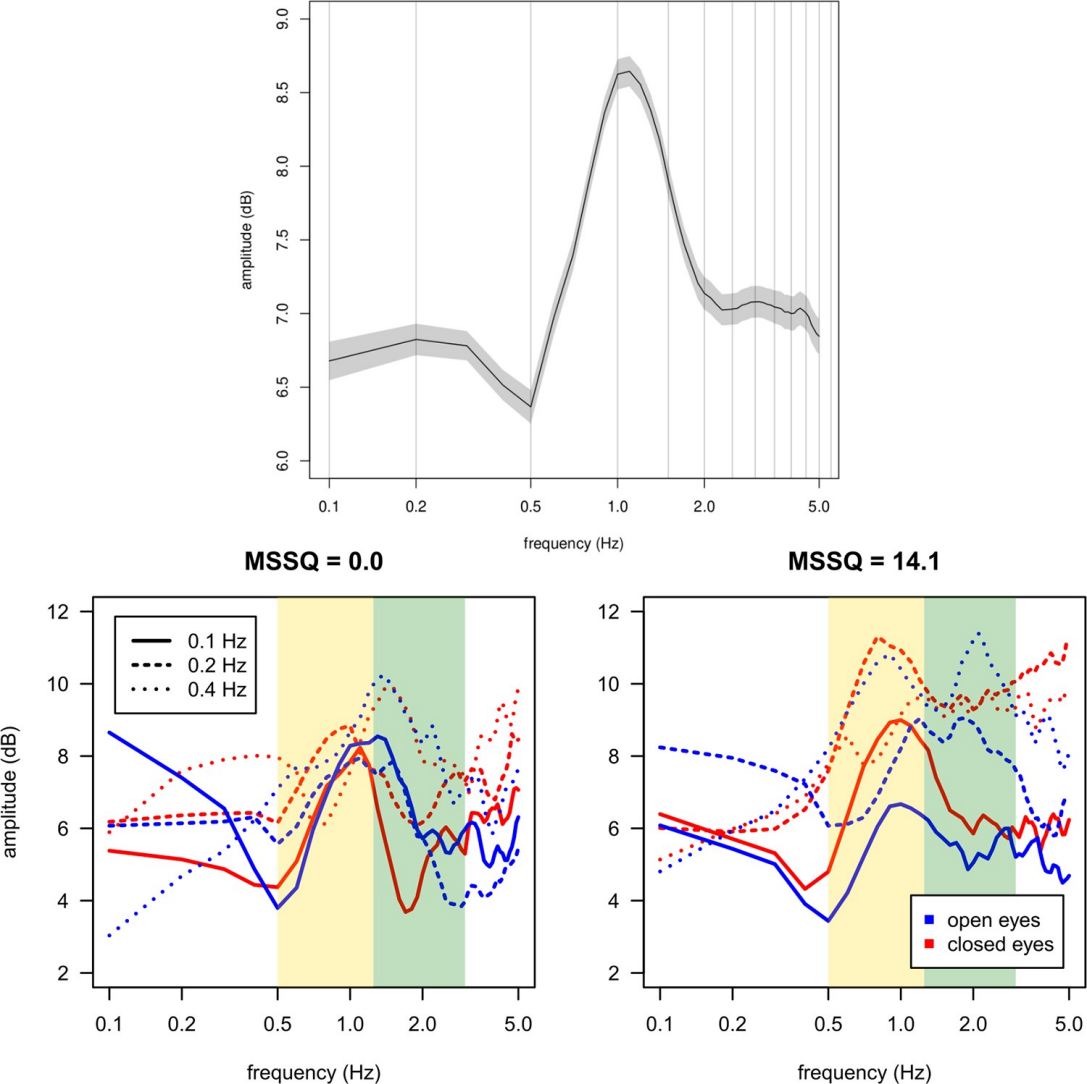
Top: Average of the PC1 eigenvectors for all participants. Bottom: Dependency of the spectral balance on the MSSQ, frequency, and visual conditions. Left panel: Examples of PC1 for selected participants with little to no differences in spectral bump between EO and EC (left) and greater differences (right). The eigenvectors for each case are shown in blue for the EO condition and in red for the EC conditions. Different types of lines were used for the different perturbation frequencies with the continuous one for 0.1 Hz, the dashed one for 0.2 Hz and the dotted one for 0.4 Hz. The yellow and green vertical stripes correspond to the frequency bands used for computing the spectral balance score.

In order to assess the spectral characteristics of the posturographic signal, we analyzed the eigenvectors associated with the PC1. This eigenvector corresponds to the variation of the postural spectrum between the perturbation phase, on one side, and the baseline and return phases taken together, on the other side. Fig 3 shows the eigenvectors for two representative subjects. We calculated the spectral energy differences between two frequency bands, one rather low frequencies, from 0.5 to 1.2 Hz and the other, higher frequencies, from 1.2 to 3.0 Hz. These two frequency regions are indicated as the yellow and green bands, respectively, in Fig 3. The threshold value of 1.2 Hz was chosen because the mean of the eigenvector obtained for all trials is characterized by a general peak around this frequency (see Fig 3 top panel). The regions below 0.5 Hz and above 3 Hz present a spectral amplitude at least 1.5 dB below the maximum value. We then computed the mean amplitude values for these low (L) and high (H) frequency regions. The spectral balance is then obtained as the difference H–L. Hence, for each subject, each visual condition, and each perturbation frequency, a value was computed for the spectral balance. Positive values of this difference therefore mean that there is more energy in the “high” frequencies (above 1.2 Hz) during the trial and negative values mean that there is more energy in the low frequencies (below 1.2 Hz). The effects of these spectral energy differences were obtained using a linear statistical model setting the visual condition and the oscillation frequency as a discrete fixed factor and the MSSQ as a continuous fixed factor.

#### Temporal characteristics of the PC1

In order to assess the temporal characteristics of the PC1, we quantified the adaptation to the perturbation and the re-adaptation during the return phase. To do so, we have fitted the following exponential model to the temporal evolution of the PC1:

$$y=A+(B-A)e^{-C(t-t_{0})}$$

where *y* is the PC1 score, *t* is the time, *t*_*0*_ is the initial time (65 s for the perturbation phase and 125 s for the return phase), *A* is the asymptotic value for *t* → ∞, *B* is the value of for *t = t*_*0*_, and the slope *C* whih is the inverse of the time constant τ (the amount of time that it takes for the value of y to be divided by the Euler’s number *e* ≈ 2.718). Separate fittings were done for the perturbation and the return phases. We used the nls function of the R software [37] for doing the non-linear least squares fitting. The value of *A* was assumed to be constant across conditions, while *B* and *C* were allowed to vary between the EO and EC conditions, as well as across the three perturbation frequencies. We also consider a linear dependency of parameters *B* and *C* with the order of the trials. Details of the exponential model fitting and results for the baseline phase in S3 Fig.

By introducing specific parameters for each frequency condition, for each viewing condition, and for the order of the blocks, we were able to highlight the effects of these factors on the coefficients, and of the model. Then, we calculated for each trial (a given participant, visual condition and frequency) the lag score defined as the tendency to be late or early with respect to the curve predicted by the exponential model. This variable is calculated as the mean of the difference between the PC1 curve of the trial in question and the predicted curve, divided by its standard deviation. A mixed-effects linear model was fitted to the data using the lmer function of R [37], in which the visual condition was a fixed, discrete factor, and the MSS score was considered as a fixed, continuous factor. The intercept value for the participant was taken as a random factor.

## Results

### Spectral characteristics of the PC1

**Fig 4:**
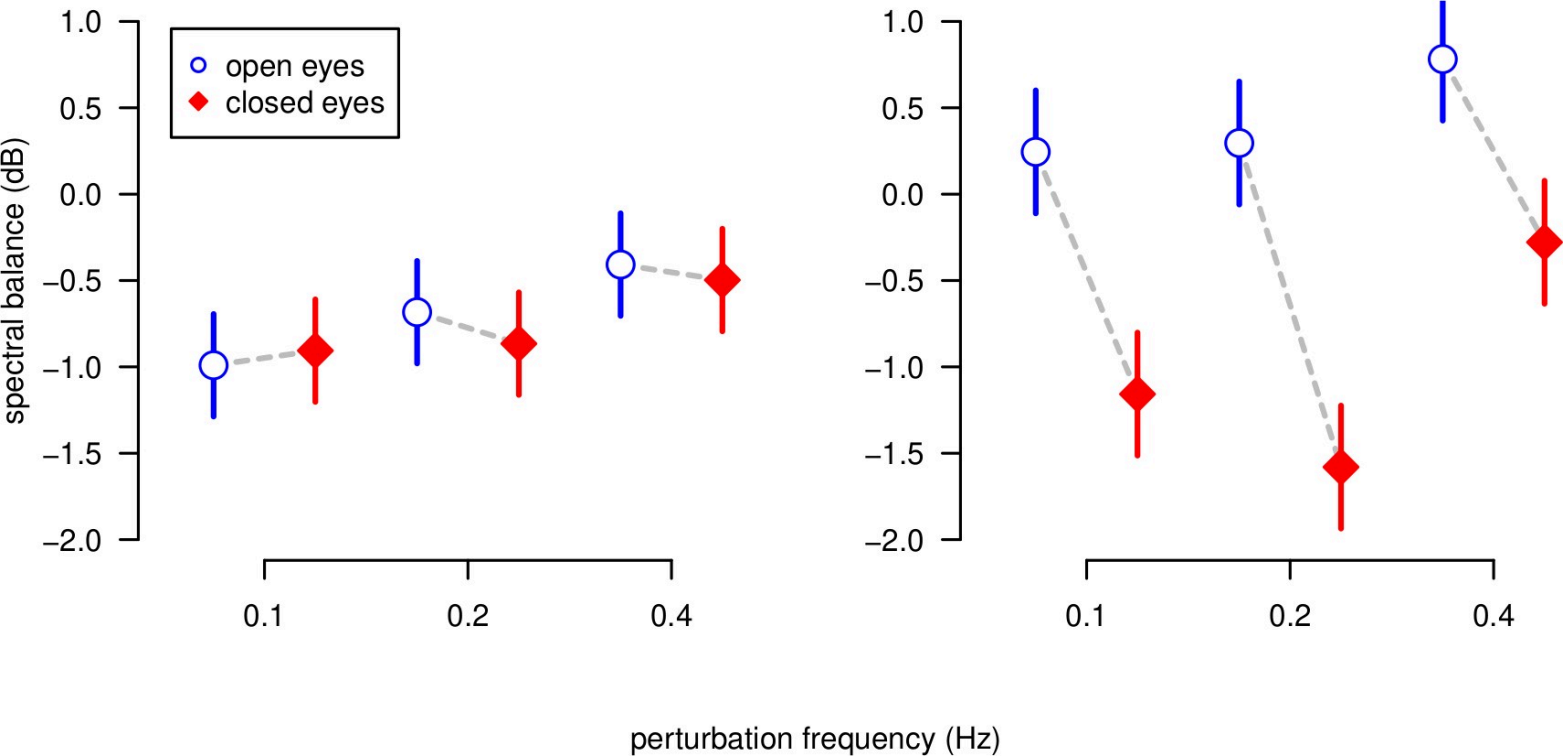
Spectral balance values predicted by the mixed-effects linear model. The values predicted for MSSQ = 0 (no susceptibility to motion sickness) are shown on the left panel and ones predicted for MSSQ = 14 (high susceptibility to motion sickness) on the right panel. EO cases are shown as open squares and EC as solid circles.

The vision condition has a significant effect on the spectral balance (*F*[1,252] = 27.7, *p* < 0.001), with the spectral balance in the EO condition higher than in the EC condition. There was a significant effect of the perturbation frequency (*F*[1,252] = 9.22, *p* < 0.001). Post-host contrast tests showed that the spectral balance does not vary between the 0.1 Hz and 0.2 Hz perturbation conditions (*p* > 0.92) but the spectral balance for the 0.4 Hz condition was higher than the mean for the other two frequency conditions (*t*[252] = -4.292, *p* < 0.001). There was no significant effect of the MSS score on the spectral balance. However, the interaction effect vision × MSS score was significant (*F*[1,252] = 8.90, *p* < 0.01). As shown on Fig 4, participants with low MSS scores had a similar spectral balance in the EO and EC conditions. In contrast, participants with high MSS scores showed a decrease of the spectral balance in the EC condition.

### Temporal characteristics of the PC1

**Fig 5:**
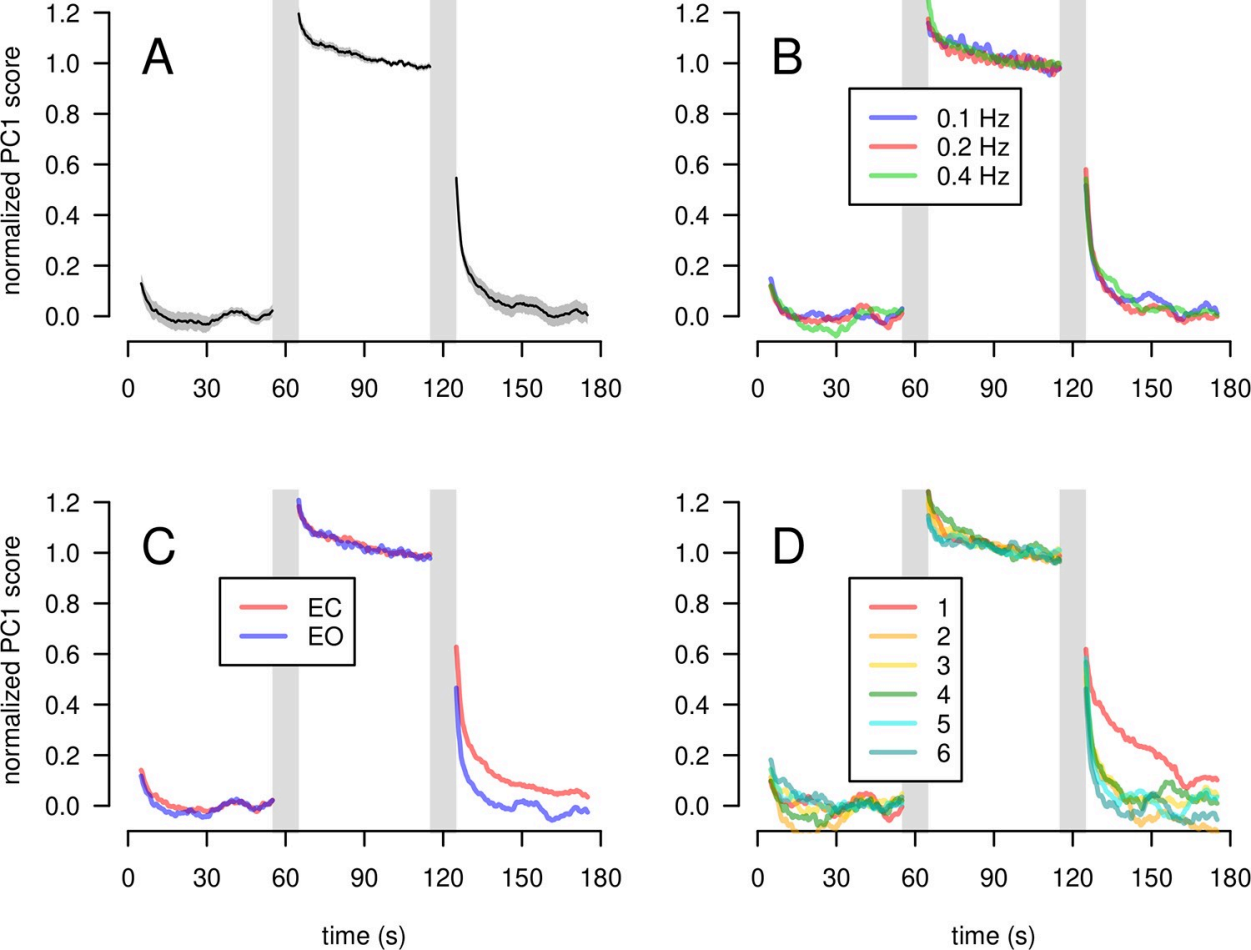
Averaged time course of the normalized PC1. The vertical gray bars depict transitional intervals that do not correspond to a definite experimental condition (10s-window spanning the adjacent baseline, perturbation, and return phases). A: Population-average. The mean value is shown by the black line. The gray band corresponds to the 95% confidence interval. B: Population-average for each perturbation frequency. C: Average across participants for each visual condition. D: Population-average for each block order.

Fig 5A illustrates the normalized PC1 score averaged across all trials. It is possible to observe an exponential behavior of the normalized PC1 scores curves, with an overshoot in the perturbation phase and a gradual return to the baseline value (zero) in the return phase. We notice a clear effect of the visual condition in the return phase. Indeed, participants tend to restore their baseline behavior more slowly in the EC condition than in the EO condition (Fig 5C).

For the perturbation phase, the value of *A*, the asymptotic value for *t* → ∞, was close to 1 (95% CI [0.98, 0.99]), the value of *B*, the value for *t = t*_*0*_, was significantly greater than 1 (95% CI [1.16, 1.20]), and the value of *C* (the slope) was positive (95% CI [0.055, 0.081]). These results suggest that the participants attained a stationary spectrum during the perturbation phase, at least during the time interval 90 to 115 s that was used in the normalization procedure. The visual condition had no significant effect on the value of B or C.

For the return phase, the value of *A* was not significantly different from zero (95% CI [-0.0051, 0.0080]). This demonstrates that the subjects reached a stationary state in the return phase. The value of *B* was around 0.37 for the EO condition and around 0.56 for the EC condition. This increase was significant (95% confidence interval [0.15, 0.24]) which shows that the postural spectrum achieved during the perturbation phase persists at the beginning of the return phase more in the EC condition than in the EO condition. The value of *C* was around 0.050 for the EO condition, with a significant decrease of –0.021 in the EC condition (95% CI [–0.038, –0.01]). These values correspond to time constants of τ ≈ 20s for the EO and τ ≈ 35 s for the EC condition, indicating that participants returned faster to their baseline sway pattern with EO than with EC.

**Fig 6:**
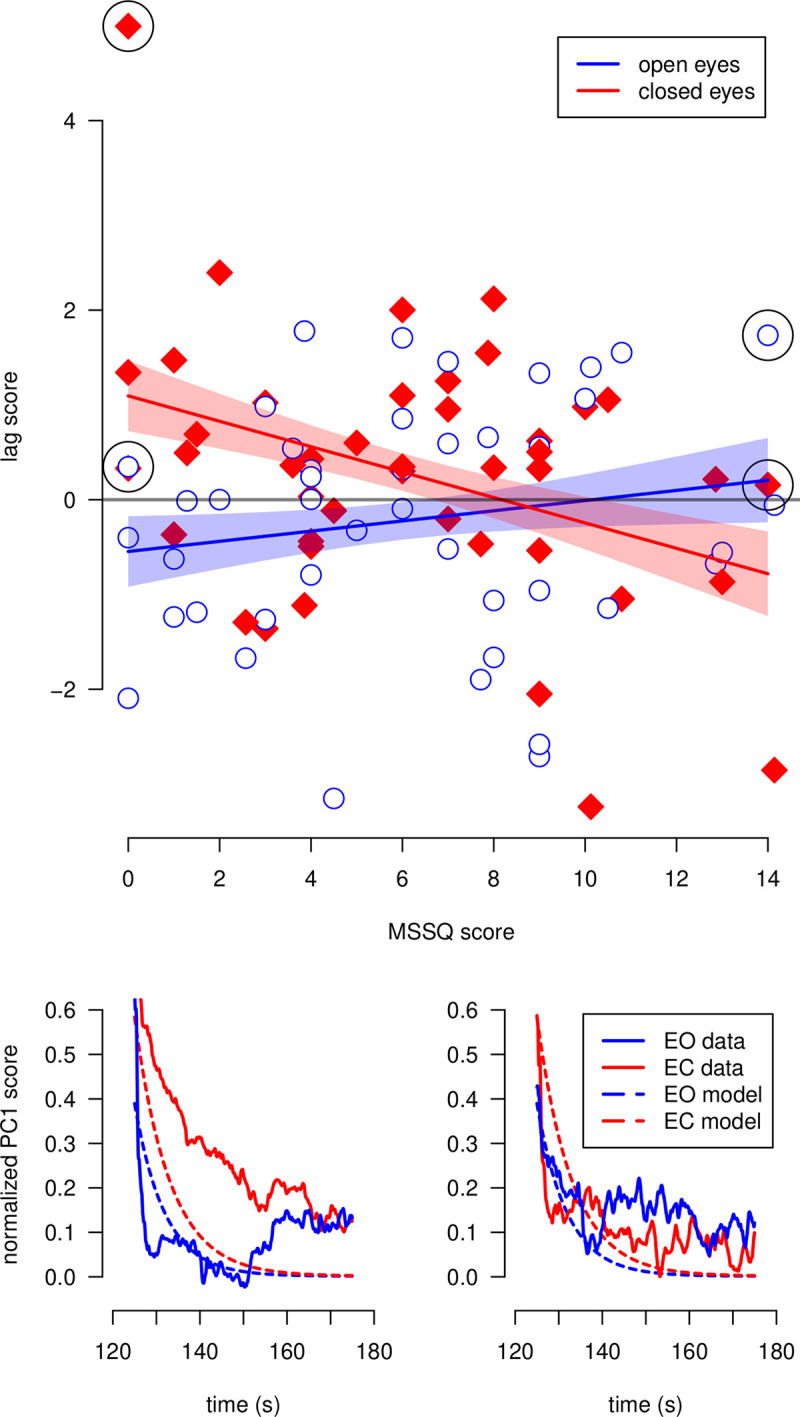
Dependency of the lag score for the return phase on the MSSQ score. On the bottom panel, examples for two selected participants are shown. The participant on the left side has a MSSQ score equal to zero, whereas the MSSQ score of the participant on the right side is equal to 14. The trajectories of the normalized PC1 score for the return phase with EO and EC are indicated with blue and red solid lines, respectively. The trajectories predicted by the exponential model for both vision conditions are represented by dotted lines. On the top panel, the scatter plot of the lag score against the MSSQ score is shown. Each point represents a participant in a given vision condition (open blue circles: open eyes, closed red diamonds: closed eyes). The points surrounded by black circles correspond to the two participants on the bottom panel. Regression lines predicted by the linear model are shown with blue and red lines, for EO and closed, respectively. 95% confidence intervals for the model predictions are shown in colored bands.

As mentioned above, we calculated a lag score for each trial, defined as the tendency to be late or early to come back to initial oscillations after the perturbation. Large interindividual differences were observed, with participants showing positive and negative lags in respect to the exponential curve predicted by the model. The behavior of two representative participants are shown in the lower panel of Fig 6, with a positive lag score depicted in the bottom left panel and a negative lag score depicted in the bottom right panel. Each point in this figure corresponds to the lag score averaged across the three perturbation frequencies. The higher this score, the slower is the return to the baseline, and vice-versa. The MSS score is represented in the horizontal axis of Fig 6 (top panel). The regression lines predicted by the model are shown in Fig 6 (top panel), with their corresponding 95% confidence intervals. The slope for the EO condition is not significantly different from zero, meaning that, with full vision, participants have essentially the same behavior, regardless of their MSS score. However, for the EC condition, a significant negative slope was found. In this condition, participants with low MSS score (low susceptibility to motion sickness), tend to return more slowly to the baseline level than participants with high MSS score (high motion sickness susceptibility).

## Discussion

### Combination of PSD and PCA for posturographic analysis

In the present study, posture was characterized by the PSD of the anteroposterior component of the CoP. The PSD was computed continuously in a sliding 10 s time window over the three phases (baseline, perturbation, and return) in each block. The change over time in the PSD spectra was captured as the first component of the PCA applied to the block data. Even though frequency spectra are widely used in posturographic research, the data processing technique used in the present study (a combination of PSD with PCA) is innovative. The reason for this choice was twofold. Firstly, we needed a posturographic measurement that made sense for short periods of time (i.e. for a 10 s time window, in our case). This requirement ruled out some conventional metrics used in similar studies, such as the maximum CoP amplitude sway, total CoP path [9], variability, velocity, range [38], recurrence quantification analysis [39]), and cross-spectral coherence [16, 40]. All these measurements only make sense when applied to broad time windows or even for the whole duration of each phase in each experimental block. Another possible measurement is the postural time-to-contact [41], which is indeed an instantaneous quantity. However, this measurement does not produce smooth values over time, let alone the kind of asymptotic behavior observed with the PC1 of the PSD in our experiment. Secondly, we chose the PSD-PCA combination because it yields a natural interpretation in terms of multisensory reweighting, as we explain in detail below. Note that our technique is based on the spectral measurement of the CoP signal, which is commonly used in postural analyses [10]. Compared to other posturographic measurements (such as range, velocity, or the area of CoP), spectral measurements provide a finer analysis of the oscillatory mechanisms underlying postural control. Furthermore, the new technique introduced in this study allows the analysis of the temporal evolution of the postural spectrum.

### Multisensory reweighting

This experiment showed that the adaptation and de-adaptation to postural perturbations (i.e. oscillation of the supporting platform) depended on the visual conditions. This result can be interpreted in the light of the neurophysiological model published by Peterka et al. [23, 28], which predicts the impact of multisensory reorganization on the body sway spectrum during postural control. Our present results showed that the grand average of the PC1 eigenvalue had a peak above 1 Hz. This finding suggests that our study participants increased the total gain of their feedback control system in order to remain stable during movement of the platform. In Peterka’s model, this peak can be interpreted in two ways. On one hand, participants might overproduce corrective torque because the sum of the sensory modality weights (W) is greater than one (i.e. overweighting). A situation like this could arise at times when the provision of sensory information is suddenly restored and the system does not decrease the associated weighting (previously increased to cope with the lack of information) rapidly enough. On the other hand, the spectral peak and the overproduction of corrective torque might be due to a simple increase in the gain in the model’s PID controller. As we have seen, the vision condition had a significant effect on the spectral balance. Indeed, the spectral balance was higher in the EO condition than in the EC condition; there was more energy in frequencies above 1.2 Hz in the EO condition than in the EC condition. In light of Peterka’s model [28], this suggests that on average, our participants tended to overweight sensory information during the experiment—probably because of the swaying platform.

The fact that postural stability in the present study was lower in the EC condition than in the EO condition confirms the impact of vision on this variable. However, the mechanisms by which vision operates are still subject to debate, and the relationship between vision and postural control is unlikely to be unidirectional. Although ocular and extraocular signals can provide essential cues for stabilizing the body in space (for a review, see [42]), stance can sometimes, be maintained and adjusted to suit the visual task [43, 44]. For instance, asking participants to perform a precision aiming task with a handheld laser pointer controlled by the postural system results in a reduction of postural oscillations in the direction related to the precision task [43]. Furthermore, the reduction in oscillation increases as the distance from the target increases [43]. On the same lines, Stoffregen et al. [45] showed that body sway was lower when participants were engaged in a visual search task (counting the occurrence of a target letter in a text) than when they were simply inspecting the text. On the basis of our present results, it would be speculative to consider the observed visual effect as reflecting the importance of vision for postural control or the importance of postural control for the fixation task. If the second hypothesis is true, closing the eyes would free the postural system from helping to stabilize the visual system. It must be noted that gazing at a visual target (e.g. a fixation cross, as in the present experiment), has been shown to stabilize the body [46]. However, in contrast to aiming or search tasks, this stabilizing effect decreases as the eye-target distance increases [47]. This is in agreement with what would be expected if ocular or extraocular cues are involved in postural control [42] but does not completely rule out other hypotheses.

### Idiosyncratic multisensory reweighting and susceptibility to MS

Our study’s main finding was that idiosyncratic ways of adapting and de-adapting to postural perturbations are related to MS susceptibility. Our results for the dependency of the spectral balance on the interaction vision × MSS score can be interpreted according to Peterka’s model. Our linear model was fitted to the data and predicted that participants not susceptible to MS (i.e. an MSS score = 0) have similar spectral balance values in the EC and EO conditions (see the left-hand panel in Fig 4). According to Peterka’s model [7], this can only be achieved by changing the relative gains for all the sensory modalities, in order to compensate for the change in visual conditions. If, in contrast, these relative gains remain the same in the EO and EC conditions, one should observe a decrease in the spectral balance—meaning that the body sway frequency tends to fall when visual information is lost. This is exactly what the linear model predicts for our MS participants (with an MSS score of 14; see the right-hand panel in Fig 4). These differences in the spectral characteristics of the PC1 eigenvector suggest that during the trials, motion-sick and non-motion-sick participants had their own individual strategies for multisensory integration.

The temporal characteristics of the PC1 supports the latter hypothesis; we found that in the EC condition, participants with low susceptibility to MS took longer to return to their normal pattern of body sway. In contrast, participants with high susceptibility to MS appeared to de-adapt almost instantaneously–just as in the EO condition. While this finding initially appears rather counterintuitive, it can be easily interpreted by the spectral balance and postural control model discussed above. In fact, participants with a low MSS score fine-tuned the weights of the non-visual sensory modalities (as shown by the spectral balance results), in order to deal with the challenging platform perturbation in the EC condition. Once the perturbation ceases, these non-motion-sick participants might have kept this weighting pattern, and so only slowly returned to their baseline sway spectrum. In contrast, people with MS might have had trouble fine-tuning the weighting of sensory information in the EC situation—even though they almost certainly increased the overall feedback-loop gain during the perturbation phase. Once the return phase started, the participants have to reset the overall gain to its initial value. This explains why participants with a high MSS score returned faster (according to the lag score) to their baseline sway spectrum than participants with a low MSS score. Another possible explanation for the slow return to the initial oscillations for participants with low MSS score is that the return phase is probably not perceived to be a dangerous situation in which rapid adaptation is required. In fact, some researchers have evidenced time-domain asymmetry between postural control adaptation and de-adaptation; less reweighting is performed when the environment goes from challenging to easy than the other way round [29].

Many authors have hypothesized that postural stability is related to susceptibility to MS. As discussed in the Introduction, postural instability may be the cause of MS [5]. A second hypothesis suggests that MS causes postural instability [8, 48]. In the present study, we highlighted a correlation between the idiosyncratic multisensory integration strategies involved in postural control and susceptibility to MS. We therefore suggest that dynamic adaptation (through sensory reweighting) is an important factor for MS. However, our results neither contradict the postural instability theory [5] nor confirm the “neural mismatch” theory [7]. Indeed, this experiment was not designed to provoke MS symptoms or induce sensory conflicts. Thus, even before the appearance of MS symptoms, idiosyncratic multisensory integration strategies for postural control correlate with the history of susceptibility to MS.

## Conclusion

By using a principal component analysis technique that extracts the change over time in the power spectrum density of the body sway signal, we were able to infer (i) the change in the body sway spectrum (reflecting adaptation to the perturbation) and (ii) the time course of de-adaptation once the perturbing oscillation has ceased. Whereas most previous studies of the relationship between MS and postural control subjected participants to nauseogenic situations, our results highlight a relationship between impaired sensory reweighting ability and MS susceptibility in situations that do not induce symptoms of MS. Further research could focus on the extent to which these idiosyncratic reweighting strategies herald the postural instability observed in individuals susceptible to MS.

## Supporting information

S1 Fig Mean global power spectrum density (PSD) of the anterior/posterior signal computed over the whole trial. Left panel: the mean PSD across all trials is represented by the black line. The vertical lines indicated the frequencies 0.01 Hz and 5 Hz. The values of the mean PSD at those frequencies are indicated by the gray dots and the vertical lines. Right panel: histogram of the difference between the PSD levels at frequencies 0.01 Hz and 5 Hz.
(PDF)

S2 Fig Histogram of the variance explained by the first principal component (PC1). (PDF)

S3 Fig Estimated parameters for the exponential model. Upper panel: baseline phase, middle panel: perturbation phase, lower panel: return phase. The estimated values are indicated by the short vertical lines and the horizontal bars represent the 95% confidence intervals of the estimations.
(PDF)
