# Supplementary figures and images for: Idiosyncratic multisensory reweighting as the common cause for motion sickness susceptibility and adaptation to postural perturbation
Source: PLoS One. 2021 Dec 9;16(12):e0260863. doi: 10.1371/journal.pone.0260863 (PMC8659652; doi:10.1371/journal.pone.0260863)

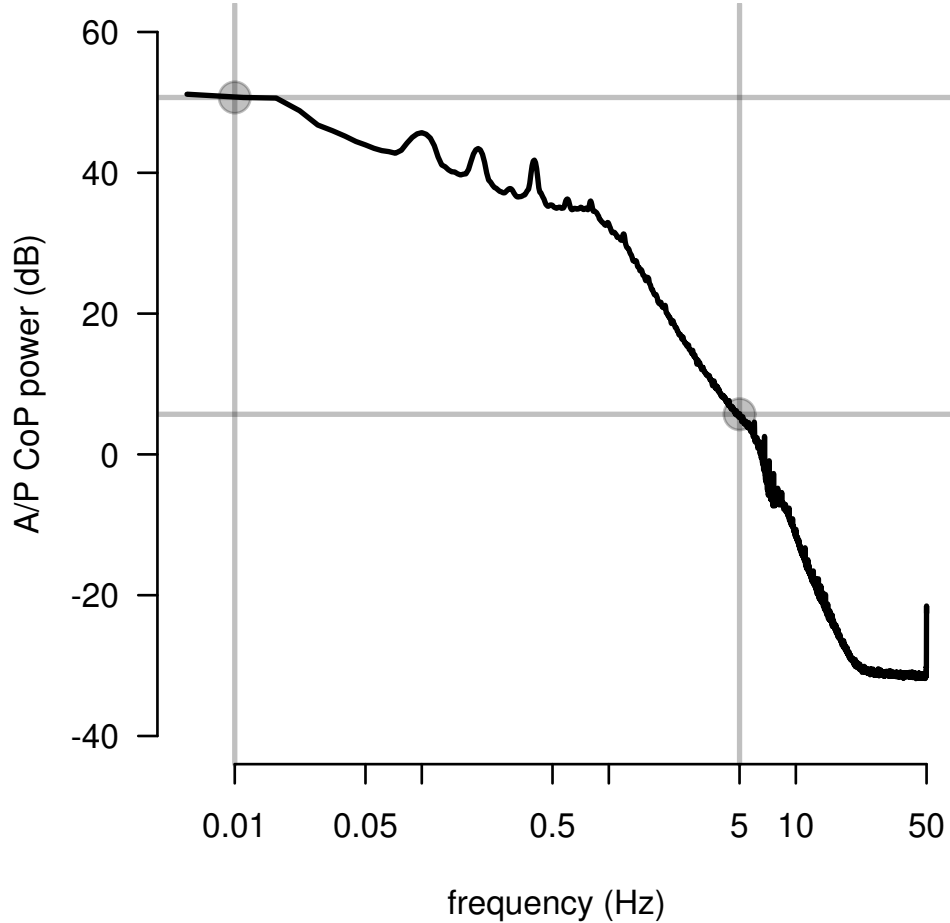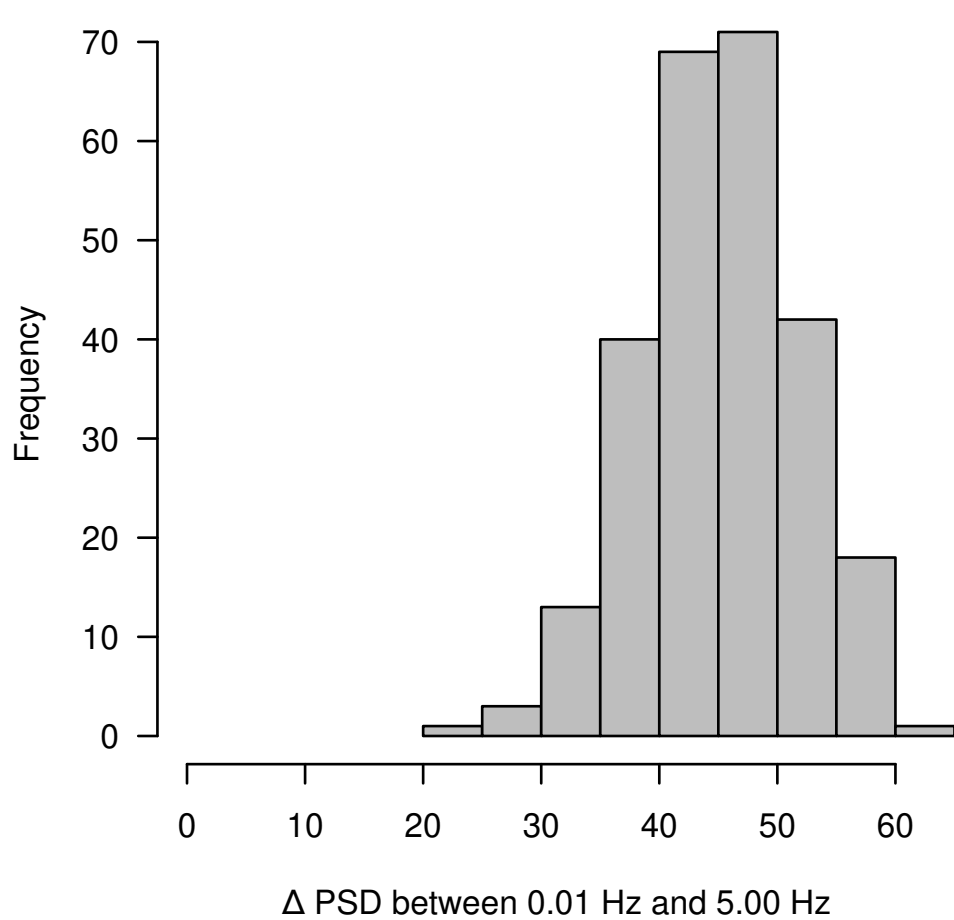

Supplement: S1 Fig — Left panel: the mean PSD across all trials is represented by the black line. The vertical lines indicated the frequencies 0.01 Hz and 5 Hz. The values of the mean PSD at those frequencies are indicated by the gray dots and the vertical lines. Right panel: histogram of the difference between the PSD levels at frequencies 0.01 Hz and 5 Hz. (PDF) [file pone.0260863.s001.pdf]

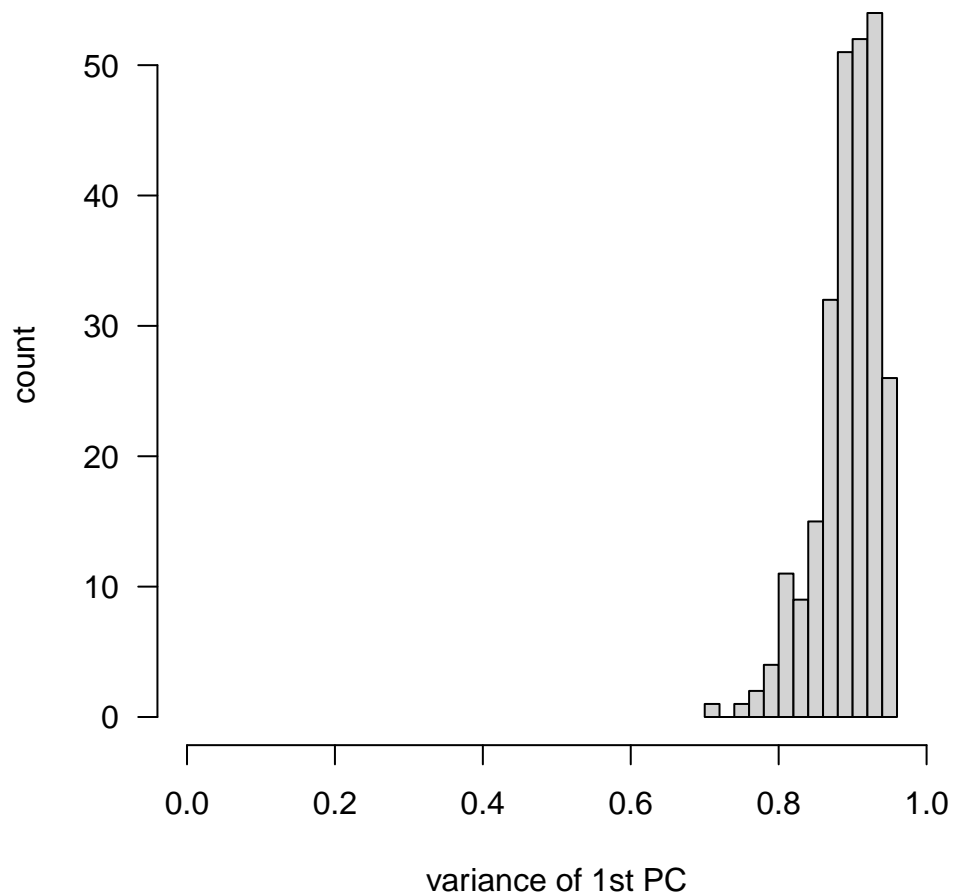

Supplement: S2 Fig — (PDF) [file pone.0260863.s002.pdf]

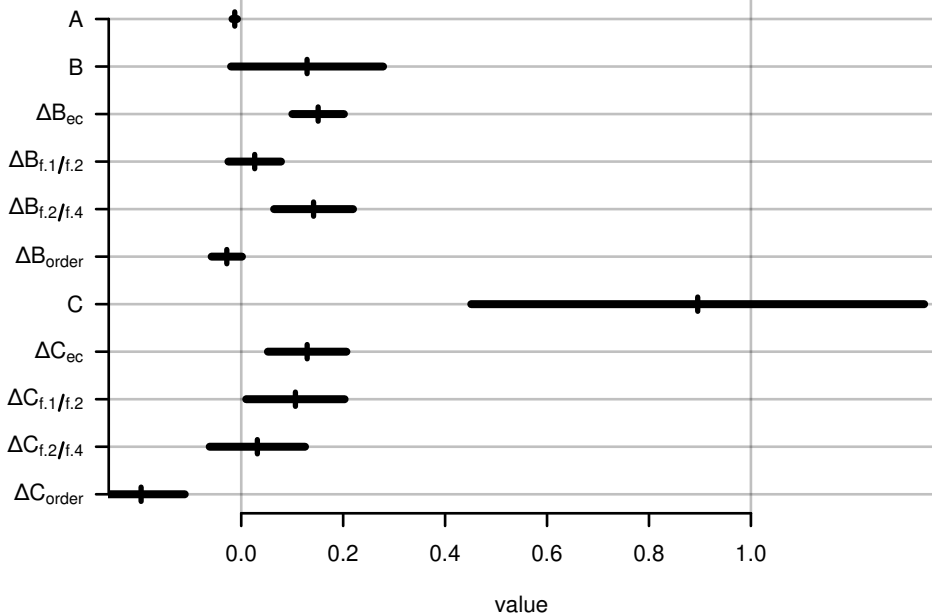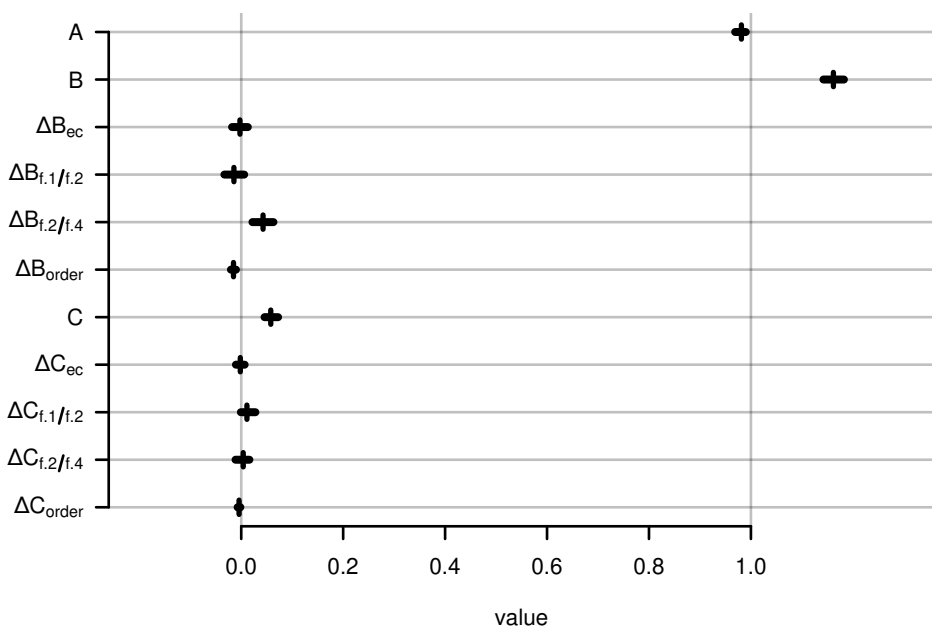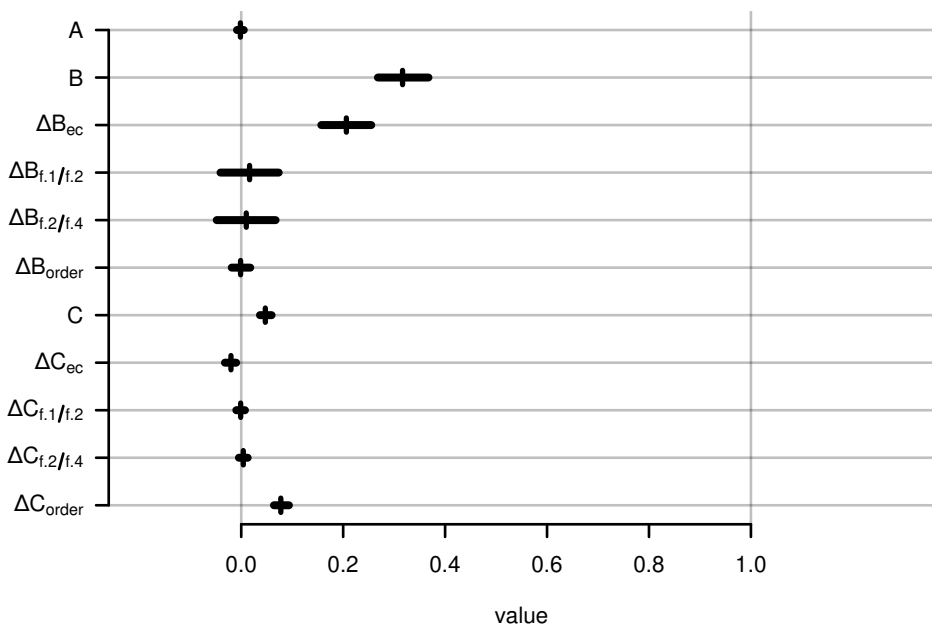

Supplement: S3 Fig — Upper panel: baseline phase, middle panel: perturbation phase, lower panel: return phase. The estimated values are indicated by the short vertical lines and the horizontal bars represent the 95% confidence intervals of the estimations. (PDF) [file pone.0260863.s003.pdf]
